# Assessment and Management of Platinum-Related Ototoxicity in Children Treated for Cancer

**DOI:** 10.3390/cancers12051266

**Published:** 2020-05-17

**Authors:** Alberto Romano, Michele Antonio Capozza, Stefano Mastrangelo, Palma Maurizi, Silvia Triarico, Rolando Rolesi, Giorgio Attinà, Anna Rita Fetoni, Antonio Ruggiero

**Affiliations:** 1 Pediatric Oncology Unit, Fondazione Policlinico Universitario A.Gemelli IRCCS, Universita’ Cattolica Sacro Cuore, 00168 Rome, Italy; albertoromano90.ar@gmail.com (A.R.); micheleantoniocapozza@gmail.com (M.A.C.); stefano.mastrangelo@unicatt.it (S.M.); palma.maurizi@unicatt.it (P.M.); silviatriarico@libero.it (S.T.); giorgio.attina@policlinicogemelli.it (G.A.); 2 Otolaryngology Division, Fondazione Policlinico Universitario A.Gemelli IRCCS, Universita’ Cattolica Sacro Cuore, 00168 Rome, Italy; rolando.rolesi@policlinicogemelli.it (R.R.); annarita.fetoni@unicatt.it (A.R.F.)

**Keywords:** children, cancer, ototoxicity, platinum compound, chemotherapy

## Abstract

Platinum compounds are a group of chemotherapeutic agents included in many pediatric and adult oncologic treatment protocols. The main platinum compounds are cisplatin, carboplatin, and oxaliplatin. Their use in clinical practice has greatly improved long-term survival of pediatric patients, but they also cause some toxic effects: ototoxicity, myelosuppression, nephrotoxicity, and neurotoxicity. Hearing damage is one of the main toxic effects of platinum compounds, and it derives from the degeneration of hair cells of the ear, which, not having self-renewal capacity, cannot reconstitute themselves. Hearing loss from platinum exposure is typically bilateral, sensorineural, and permanent, and it is caused by the same mechanisms with which platinum acts on neoplastic cells. According to available data from the literature, the optimal timing for the audiological test during and after treatment with platinum compounds is not well defined. Moreover, no substances capable of preventing the onset of hearing loss have been identified.

## 1. Introduction

Platinum compounds are a group of chemotherapeutic agents included in many pediatric and adult oncologic treatment protocols. Their use in clinical practice has greatly improved long-term survival of pediatric patients [1].

The main platinum compounds are cisplatin, carboplatin, and oxaliplatin. They exert their action by covalent binding to purine DNA bases, resulting in interference with normal DNA function [2,3].

Cisplatin was the first compound discovered in 1965 by Rosenberg and colleagues during an experiment on electric fields and cell division of bacteria, and was introduced in clinical practice in the first years of the 1970s [4]. Its cytotoxic effect is linked to the formation of cisplatin–DNA adducts (adducts N-7 a d (GpC) and d (ApG)) that block the vital functions of cancer cells with consequent death [4].

After intravenous infusion, 90% of cisplatin binds to plasma proteins and has a high capacity to penetrate into the liver, kidneys, testicles, colon, and small intestine, but does not normally pass into the central nervous system (CNS) [5].

About 90% of the drug is eliminated from the body through the kidneys, thanks to the glomerular filtration and tubular secretion processes, whereas 10% is eliminated by biliary excretion. Generally, 25% of cisplatin is eliminated from the body within 24 h after administration [2,5,6], however, platinum adducts can be found in tissues for more than a decade after exposure [7,8] or indeed for an indefinite time [9].

Chemotherapy with cisplatin is used in the treatment of several pediatric cancers, such as germ cell tumors, hepatoblastoma, medulloblastoma, medulloblastoma/primitive neuroectodermal tumor (PNET), neuroblastoma, high-risk osteosarcoma, and refractory lymphomas [7,8].

The main adverse effects associated with cisplatin are nephrotoxicity, neurotoxicity, and ototoxicity; to try to avoid them, the dose of subsequent administrations of the drug must be reduced [10,11,12,13].

Carboplatin is an analogue of cisplatin that was developed to reduce the dose-limiting toxicity of cisplatin. Carboplatin is less toxic than cisplatin because it reacts less with DNA due to its greater chemical stability [5].

Previous studies have proven that carboplatin is 8–45 times less potent than cisplatin and, to obtain a comparable antitumor effect, higher doses of carboplatin are necessary [14,15]. Lokich et al. (1998) [6] have pointed out that to obtain the same effect determined by 100 mg/m^2^ of cisplatin, a dose of 400–500 mg/m^2^ of carboplatin is required, approximately four times higher.

Carboplatin is commonly used in the treatment of pediatric tumors such as hepatoblastoma, brain tumors, germ-cell tumors, neuroblastoma, Ewing sarcoma, osteosarcoma, and relapsed or refractory Wilms tumor [16].

Veal et al. (2007) reported that about a third of all children with solid tumors receive chemotherapy with carboplatin [17]. Unlike cisplatin, carboplatin has a lower affinity for plasma proteins. Ninety percent of carboplatin is removed by the kidneys through glomerular filtration within 24 h after its administration [18].

Oxaliplatin (trans-L-diaminocyclohexane (DACH) platinum oxalate II) is a third-generation platinum compound [19,20]. Oxaliplatin has a lower affinity for DNA than cisplatin because the DACH vector ligand converts more slowly the monoadduct into a diadduct. Nevertheless, the DACH–platinum adducts are highly cytotoxic, and the DACH vector ligand prevents cells from developing mechanisms that allow them to overcome platinum toxicity [21].

In addition, oxaliplatin shows no cross resistance with cisplatin or carboplatin, and this is due to two types of mechanisms. The first one is related to the drug structure; the DACH ring is large enough to prevent the mismatch–repair enzyme complex from binding to the oxaliplatin adducts [22]. The second mechanism is due to the absence in tumor cells of the replicative bypass activity present in cisplatin-damaged cells [23].

Oxaliplatin has pharmacokinetic characteristics similar to cisplatin and carboplatin. After intravenous administration, initially about 70% of the drug binds to plasma proteins, especially albumin, and subsequently up to 95% of the drug is bound to proteins. When bounded to plasma proteins, the drug loses some of its anti-tumor activity. Furthermore, oxaliplatin is able to penetrate erythrocytes and remains there longer than in plasma. On this basis, O’Dwyer et al. (2000) reported that red blood cells can probably represent a drug reservoir [5,19].

The main route of oxaliplatin elimination is represented by the glomerular filtration of the kidney, and only 2% of it is eliminated in the feces. There is no correlation between renal function and platinum exposure (AUC). Unlike the other platinum compounds, oxaliplatin is better tolerated by the human body, and it causes hearing damage in less than 1% of the patients and renal toxicity in less than 3%. Moreover, although oxaliplatin can determine neurotoxicity affecting the peripheral sensory nerves, this is rapidly reversible because the drug does not accumulate even after multiple cycles of treatment [19].

As reported in several studies [24,25], oxaliplatin has a wide-spectrum antitumor activity against solid tumors.

Cisplatin, carboplatin, and oxaliplatin are all compounds obtained from platinum and have some similar pharmacodynamic characteristics; nevertheless, they each present specific antineoplastic activity and are therefore used in different treatment protocols for tumors both of pediatric and adult population.

## 2. Pathogenesis of Hearing Damage from Platinum Compounds

Hearing damage is one of the main toxic effects of platinum compounds. It derives from the degeneration of hair cells of the ear, which, not having self-renewal capacity, cannot reconstitute themselves [26]. Hearing loss from platinum exposure is typically bilateral, sensorineural, and permanent [27]. The higher frequencies (>4000 Hz) are the first to be affected, but damage can progressively affect even the lowest frequencies (500–4000 Hz), which are those necessary to understand language. Although hearing damage becomes evident when lower frequencies (500–4000 Hz) are affected, even the absence of high frequency audibility is important in children, as it can affect the regular development of speech.

Sometimes, hearing loss can be perpetuated even after the suspension of therapy with platinum compounds, manifesting itself after many years [27,28,29].

**Figure 1:**
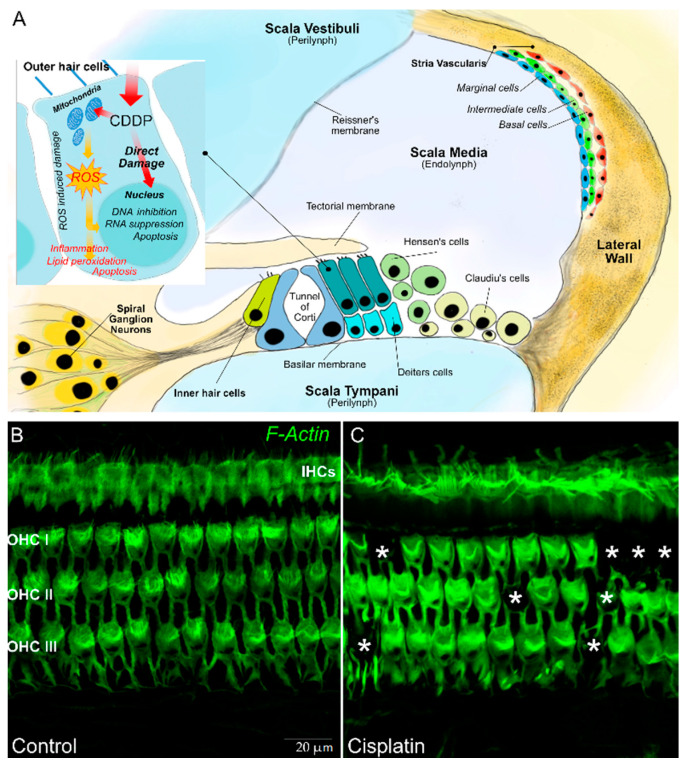
In (**A**) a schematic representation of a transverse section of the basal part of a mammalian cochlea. Organ of Corti cellular organization: one inner hair cell (IHC) and three outer hair cells (OHCs) are represented on either side of the tunnel of Corti. The tectorial membrane, floating in endolymph, caps the tallest stereocilia of the hair cells. The IHC is surrounded by supporting cells, whereas OHCs are anchored to the Deiters’ cells, their lateral membrane in direct contact with a fluid called endolymph, which fills the tunnel of Corti. The lateral wall is constituted by the stria vascularis and spiral ligament. In (**B**), representative images are shown of surface preparations of the organ of Corti stained for F actin, used to visualize the stereociliary arrays and cuticular plates of hair cells. The dark spots indicated by asterisks in (**C**) show OHC loss after a single dose of cisplatin in the rat model.

Platinum ototoxicity is due to the degeneration of mechanosensory hair cells in the organ of Corti, the spiral ganglion, and the lateral wall cells (stria vascularis and spiral ligament) [30]. About 3500 inner hair cells (IHCs) and 12,000 outer hair cells (OHCs) are contained the organ of Corti—the OHCs amplify the motion of the basilar membrane in improving low-level sensitivity and frequency selectivity, whereas the IHCs transform sound-evoked mechanical motion into receptor potentials, resulting in the release of glutamic acid by the synapses and activation of the potentials action of the cochlear afferent fibers. Spiral ganglion neurons are bipolar sensory neurons with a critical importance in the normal transmission of sound information to the brain. The stria vascularis (SV) and spiral ligament (SL) form the lateral wall of the cochlear duct. The SV is a vascular structure involved in the endolymph formation and generation of the endocochlea that are essential for mechanoelectrical transduction (Figure 1).

The mechanisms at the basis of the described damage are presumably the same with which platinum acts on neoplastic cells. The platinum compound interacts with cell DNA, inducing monoadducts at nucleophilic sites, which can subsequently lead to intrastrand and interstrand crosslinks in the DNA [31]. The resultant inhibition of DNA synthesis and of RNA transcription causes cell cycle arrest, leading to apoptosis. A secondary mechanism of cell death is linked to platinum metabolism [32]. When platinum enters the cells, it is metabolized by the mitochondria, with the consequent production of toxic levels of reactive oxygen species (ROS). ROS deplete the cell’s intrinsic antioxidant molecules and cause the destruction of proteins and lipids; this event in turn actives the caspase and cell death [33,34,35,36,37]. Hence, multiple damage mechanisms cooperate in determining the death of hair cells, including oxidative stress related to excessive production of ROS that causes lipid peroxidation, activation of pro-inflammatory factors, induction of the p53-dependent signaling pathway, and, finally, cell death by apoptosis [38].

These events can be realized only if platinum compounds cross the blood labyrinth barrier (BLB), where inner ear sensory cells reside. The passage of platinum compounds through the BLB can occur in the event that cell integrity is altered or if the paracellular permeability between adjacent endothelial cells increases. Another mechanism is provided by transport systems that include megalin (LRP2), the organic cation transporter 2 (OCT2) (SLC22A2), or the high affinity copper uptake protein 1 (Ctr1) (SLC31A1) [31,39,40].

Moreover, indirect mechanism of damage by platinum compounds on hair cell function can be caused by magnesium deficiency. Magnesium is necessary to maintain hair cell permeability [41] and cochlear blood flow [42]. During therapy, platinum compounds can damage renal tubules cells, thus impairing their reabsorption ability and reducing the intestinal absorption of nutrients. As a consequence, the plasma magnesium level can become deficient, with consensual reduction of its concentration in the endolymph [43] and perilymph [44]. This event determines an imbalance of the ionic composition of the two fluids of the labyrinth, causing an increase of hair cell permeability to platinum [41,45].

The described damage mechanisms cause hearing loss from platinum compounds, but not all children undergoing chemotherapy with these drugs show the same extent of hearing impairment. This could be explained by the existence of a genetic predisposition to platinum-induced hearing loss. In recent years, many study groups have focused their attention on the search for genes involved in platinum ototoxicity, identifying the genes involved in transport and metabolism of platinum compounds and in DNA repair as possibly responsible [31]. Among studies conducted to date, Huang et al. (2007) [46] identified 17 variations among 26 genes probably implicated in platinum ototoxicity. Among these genes, DNA damage inducible transcript 4 (DDIT4) is a mediator of reactive oxygen species generation [47]; NIMA (never in mitosis gene a)-related expressed kinase 2 (NEK2) is required for proper execution of mitosis [48]; and MYC plays a role in cell cycle progression, apoptosis, and cellular transformation; mutation of these genes could facilitate hearing loss from platinum compounds [45,49]. On the contrary, some genetic variants protect against platinum-induced ototoxicity, such as the polymorphisms in genes of the glutathione S transferase (GST) family (i.e., cisplatin detoxification and free radical scavenging) [50]. In particular, a 3-bp insertion/deletion polymorphism in intron 6 of GSTM3 (rs1799735), the c.313A > G (rs1695, p.Ile105-Val) single nucleotide polymorphism (SNP) in GSTP1, and gene deletions in GSTT1 and GSTM1 play a protective role against cisplatin ototoxicity [50,51].

Genes associated with hereditary deafness have also been studied as possible culprits genes, but their polymorphisms do not seem to be involved in hearing loss from platinum compounds [52,53].

A recent study of Clemens et al. (2020) [54] evidenced an unclear association between single genetic markers and ototoxicity on a sample of 428 cancer survivors; they studied 10 different genetic polymorphisms selected according to previous studies: ACYP (rs1872328), LRP2 (rs2075252), NFE2L2 (rs6721961), OTOS (rs2291767), TPMT (rs12201199), SOD2 (rs4880), SLC22A2 (rs316019), GSTP1 (rs1695), ABCC3 (rs1051640), and SLC16A5 (rs4788863). None of these showed a clear relation with ototoxicity, but the authors suggest that more than a single gene variation could interact in determining a predisposition to platinum hearing loss.

Hearing loss from platinum compounds is also different according to the total dose and the type of compound used. Cisplatin is the most ototoxic platinum compound, whereas oxaliplatin is the least ototoxic. In the pediatric age group, a cumulative cisplatin exposure exceeding 400 mg/m^2^ is considered the threshold for significant ototoxicity [1,45], whereas in adult patients it is 600 mg/m^2^ [1]. In fact, children under five years of age have the highest risk of suffering hearing loss after platinum treatment [55]. For carboplatin, ototoxicity has been reported to occur at cumulative doses in excess of 400 mg/m^2^ [45].

In addition to the total dose administered, the mode of administration may also facilitate platinum acoustic damage. Bolus infusions are more ototoxic compared to short or continuous infusions [56]. Up to now, there is no evidence that continuous infusions are less ototoxic than short infusions [56]. Moreover, administration of cisplatin while there is noise in the room facilitates hearing loss [3] because noise exposure can damage the stria vascularis and disrupt the BLB [57]. Noise also makes the cochlea more vulnerable because it induces oxidative stress and decreases antioxidant enzyme levels [58,59].

Other risk factors involved in hearing loss caused by platinum compounds are represented by tumor site, concomitant CNS irradiation, and the association with other ototoxic medications.

Central nervous system tumor site is associated with the highest rate of hearing loss. This high incidence is conditioned by several factors, among which is the higher dose of platinum compounds used in this type of tumor and the direct damage the tumor can cause in this area [60], particularly when chemotherapy is combined with radiotherapy [61]. Due to the presence of these hearing damage enhancing factors, the real prevalence of ototoxicity in children with brain tumors cannot be correctly estimated and, as observed by Bass et al. (2014), it can reach values of up to 74% in patients undergoing combined treatment with cisplatin and radiotherapy [62].

As previously mentioned, platinum compounds are eliminated by the renal system, and impaired renal function may delay excretion of platinum agents facilitating hearing impairment [45]. Medications such as aminoglycoside antibiotics and loop diuretics can also contribute to ototoxicity [45]. Aminoglycosides act directly by damaging the inner hair cells, reducing ROS elimination and altering the stria vascularis, facilitating platinum passage through the BLB [63]. Instead, loop diuretics reversibly block Na–K–Cl cotransporter expressed in the inner ear, altering the ionic composition of endolymph and reducing blood flow with impairment of the barrier function of the endothelium. This in turn facilitates the transition of other ototoxic drugs, such as cisplatin and aminoglycosides, into the inner ear [64], exacerbating hearing loss.

Presence of co-existing ear pathology such as chronic otitis, middle ear effusion, or cerumen impaction can further worsen auditory impairment [45].

## 3. Assessment of the Hearing Function during Treatment with Platinum Compounds

A complete evaluation of the auditory function is necessary before starting treatment with platinum compounds, as it allows for the obtaining of a basal assessment that can be compared to subsequent evaluations, during treatment and after the end of it. The baseline assessment can be undertaken with different audiological tests in relation to patient’s age.

Audiometry is the easiest test to use and allows for the detection of the minimum audibility threshold of the patient. A complete audiometric exam includes pure tone audiometry and speech audiometry [64].

Pure tone audiometry allows for the identification of the auditory threshold, the minimum intensity of a sound that the subject is able to sense. It consists of administering to the patient, in an environment free of noise pollution, tones with an increasing frequency and then asking the subject to signal the presence of a sound through a behavioral response. This type of test requires collaboration from the patient, and therefore is strongly influenced by his or her age, and thus can only be used in children over 7–8 years of age. In younger children, it is necessary to use alternative methods [26]. Children aged between 24 months and 6 years may have difficulties in performing the conventional audiometric examination, and thus they are generally evaluated with conditional audiometry, in which the child is asked to perform a simple action (such as putting an object in a container) whenever he hears a noise. Visual reinforcement audiometry is used for children aged 7 months to 24–30 months [26].

Speech audiometry, on the other hand, allows clinicians to evaluate the patient’s ability to understand spoken language, and consists of asking the patient to repeat specific words. In younger children, speech audiometry can be carried out by asking them to indicate an object or a drawing that corresponds to the communicated word. This type of test does not allow for the evaluation of hearing damage in the initial phases, which typically involves high frequencies, but allows us to estimate its impact on the child’s daily life [26].

Pure tone audiometry typically includes the frequency range of 0.25 to 8 kHz. A further form of audiometry, which can be useful in the early diagnosis of hearing damage from platinum compounds, is represented by high frequency audiometry. It is an audiometry that evaluates the hearing threshold for sounds with a frequency higher than 8 kHz and up to a maximum of 20 kHz [65]. Because auditory damage from platinum compounds occurs first for high frequencies, the use of high frequency audiometry is a useful tool for its early diagnosis. Furthermore, although the audibility of high frequencies is not involved in the understanding of spoken language, recent publications have shown that it is involved in learning phonemes during early childhood. Because early auditory damage can condition the normal development of language [66], the use of high frequency audiometry has an important role in the pediatric age.

As already mentioned, the acoustic damage from platinum compounds is sensorineural and not conductive. Therefore, before performing the audiometry test it is useful to exclude the presence of pathologies affecting the structures of the external ear through the execution of a tympanogram. This will make it possible to eliminate any confounding factors in estimating the auditory function.

In children younger than 7–8 months, audiometry is not applicable. At this age, the otoacoustic emissions (OAEs) measurement could be useful to evaluate normal ear function. OAEs measurement specifically evaluates cochlear outer hair cell function. Differently from pure tone audiometry, OAEs cannot estimate severity of hearing loss. When OAEs are altered, it is possible to use a second line test, which is the auditory brainstem response (ABR). This test allows for the estimation of the normal function of all auditory structures by measuring the transmission of the nerve impulse generated by the administration of sounds, and is applicable when behavioral testing is not possible due to young age, uncooperation, or medical conditions [67]. Table 1 resumes audiological tests and their application age.

After having obtained a basal assessment of auditory function, it is necessary to repeat it during treatment. The frequency of the tests is strongly connected to the cumulative doses of platinum administered and to the presence of other risk factors. At present, there is not much scientific evidence regarding the best time to repeat audiological tests during treatment. According to Durrant et al. (2009), follow-up evaluations should be performed 24 h prior to each course of platinum-based chemotherapy [64,67].

In our clinical practice, we perform an audiometry test before each course of chemotherapy containing platinum compounds and whenever there is a clinical sign of hearing impairment, even though it is not always possible due to patients’ clinical conditions. This strategy allows us to identify the onset of hearing damage earlier on, thus giving us the ability to carry out measures to reduce its extent.

Different evaluation scales of hearing damage caused by chemotherapy have been standardized. Among these are the National Cancer Institute Common Terminology Criteria for Adverse Events (NCI-CTCAE), the American Speech-Language Hearing Association (ASHA) ototoxicity criteria, the Brock criteria, Chang and Chinosornvatana’s grading system, and the New International Society of Pediatric Oncology (SIOP) Boston Ototoxicity Grading Scale.

The Brock criteria was the first and most widely used pediatric-specific ototoxicity scale. This scale is based on absolute hearing thresholds unrelated to the baseline test. It has four grades (1–4) to categorize the severity of platinum induced hearing loss [68].

Chang and Chinosornvatana’s grading system is a modified Brock scale to include both 20 and 40 dB HL cut-offs; it is complicated to apply and requires additional threshold data that may be difficult to obtain in an ill or uncooperative patient [68].

The National Cancer Institute Common Terminology Criteria for Adverse Events (NCI-CTCAE) has been typically used in cancer treatment studies; this rating scale, in its latest version of 2017, specifically considers pediatric patients and distinguishes five degrees of severity of hearing damage with a cut-off of 20 dB HL.

The American Speech-Language Hearing Association (ASHA) ototoxicity criteria can be used for extended high frequencies (>8000 Hz) and allows for the monitoring of the auditory threshold during treatment. In addition, these criteria require a complete baseline assessment, which is difficult to obtain in children; moreover, the major limitation of the ASHA criteria is the lack of a grading scale to evaluate the severity of hearing loss [3].

The New International Society of Pediatric Oncology (SIOP) Boston Ototoxicity Grading Scale takes into consideration the possibility that the child may present with middle ear disease (rather common in children), and requires bone conduction thresholds in case a problem with sound conduction through the middle ear is suspected, or in the presence of abnormal tympanometry. The scale is based on absolute thresholds and uses 20 and 40 dB HL cut-off, with greater importance given to hearing loss in the medium frequencies compared to the high frequencies [58].

This scale of evaluation for hearing loss is useful in clinical practice in identifying the severity of the damage in order to undertake possible protection strategies. Knight et al. (2017) compared different ototoxicity classification systems and evidenced that the SIOP scale may be superior to ASHA, Brock, and CTCAE scales for classifying ototoxicity in pediatric patients who were treated with cisplatin [69].

## 4. Audiological Follow-Up after Therapy with Platinum Compounds

At the end of treatment with platinum compounds, every patient has to be tested for hearing loss.

As previously mentioned, ototoxicity is proportionately higher in survivors treated with high cumulative cisplatin doses than with low doses and in survivors treated with platinum-based drugs who were younger at the time of diagnosis [66,67,68,70].

Recently, Clemens et al. (2019) systematically reviewed the scientific literature available to date, and drafted a document that outlines the necessary steps in the follow-up of patients undergoing chemotherapy protocols that include platinum compounds [71].

In this work, they stated that according to the published available data, it is not possible to define a surveillance time interval during which patients have to be tested. In particular, it is unclear how long-term survivors who have no hearing loss at the end of treatment should undergo hearing assessment tests [71].

According to data available in the literature, surveillance in survivors should begin at the end of treatment and should be performed annually for children under the age of 6 years, every 2 years for children aged between 6 and 12 years, and every 5 years for teenagers and young adults over the age of 12. This long surveillance is important due to the possible appearance of hearing damage, even long after the end of treatment [71].

Surveillance must be carried out with closer controls until completion of language development, and for this reason the indications of Clemens et al. (2019) provide for an annual check up to 6 years of age [71].

Usually, survivors are tested at frequencies of 8000 Hz or less and, if no losses of more than 15 dB are measured, hearing function is considered to be unaffected and surveillance is discontinued.

For routine surveillance of cancer survivors aged 6 years or older, pure-tone audiometry at 1000–8000 Hz is the gold standard. If equipment is available, additional testing with high-frequency audiometry at more than 8000 Hz is also recommended for its usefulness in the pediatric age [71].

The use of high-frequency audiometry increases the number of diagnoses of asymptomatic hearing loss in children treated with cisplatin-based chemotherapy [72].

In our experience, monitoring of the auditory function during platinum-derived treatment significantly improved our ability to detect patients with early hearing impairment. With reference to a previous work [73], our protocol consists of evaluations at the following time points: at baseline (within 2 weeks of treatment initiation) and prior to each chemotherapy cycle. After the end of treatment, the audiological long-term follow-up is performed at least once per year for 5 years, according to disease status and treatment received. The overall incidence of hearing loss was 25% in children treated with cisplatin, 19% in those treated with carboplatin, and 35% in those treated with the cisplatin–carboplatin combination. Only 13 children (12.5%) experienced sensorineural grade 2 hearing loss or more, according to the Boston SIOP ototoxicity scale. A further progression of hearing impairment after the end of chemotherapy was observed in 8.6% of children. The low rate of ototoxicity as compared to the literature suggests the pivotal role of auditory monitoring in children treated with platinum compounds. Close monitoring, which involves pediatric oncologists and audiologists/otolaryngologists, is essential to identify hearing loss at an early stage, as well as to provide strategies to reduce further progression of cochlear toxicity [73].

Education, amplification or hearing-assistive technology, cochlear implantation, hearing aids, tactile aid, frequency-modulated system, communication approaches, or intervention programs (such as early and consistent speech therapy) are all strongly recommended by Clemens et al. (2019) to minimize the social and intellectual impact of hearing loss in children [71,74,75,76,77,78,79,80,81].

## 5. Strategies of Prevention of Hearing Loss

The prevention of hearing damage is certainly a challenge for all those who deal with childhood cancer. The integrity of the auditory function is a fundamental prerequisite for the development of language and in general for the psychological and social maturation of the individual. Thus, the development of effective strategies is a major goal to protect or rescue auditory function from platinum-induced ototoxicity without affecting or decreasing the antitumor activity of these compounds. Several preclinical studies using both the transtympanic route and systemic administration indicate that antioxidant molecules, such as tiopronin, Vitamin E, curcumin and others, can rescue reactive oxygen species and improve hearing [30,82,83,84]; however, only a few clinical trials have recently been completed, with controversial results [31]. Furthermore, systemic administration of chemoprotectants is affected by the risk of chemical interaction with anticancer activity. On the other hand, hypothetical advantages of local application are undermined by the invasiveness of the therapeutic approach and the poor control of drug release, especially in children. The transtympanic administration of dexamethasone, *N*-acetylcysteine, sodium thiosulfate, or other antioxidants has been evaluated in adult clinical trials without conclusive results, as reported in the U.S. clinical trials website (http://www.clinicaltrials.gov). Therefore, in the past, some treatment protocols have considered the possibility, in the event of hearing loss, of reducing the dose of cisplatin or replacing it with a less ototoxic compound such as carboplatin [82]. However, this strategy only partially reduces the risk of suffering a hearing loss and could jeopardize treatment efficacy on the tumor.

Amifostine [85] has been one of the most studied molecules in clinical trials. It is a prodrug whose function is activated following the dephosphorylation by alkaline phosphatase at tissue level. In this way, a pharmacologically active metabolite is obtained whose name is WR-1065. This substance has the ability to bind and eliminate toxic cisplatin metabolites, and also acts as a scavenger for ROS. This activity takes place mainly in healthy tissues due to presence of a greater expression of alkaline phosphatase, a greater degree of vascularization, and a more alkaline pH [81]. At present, this compound is used with cisplatin as a nephroprotectant, but there are no studies that have confirmed its effectiveness in protecting the ear, and therefore its use has not entered into clinical practice [86,87,88].

Another substance that acts in a similar way to amifostine, by binding derivatives of platinum compounds and acting as a ROS scavenger, is sodium thiosulfate [89]. This has been tested in pediatric and adult patients with different types of tumors and has shown significant benefits compared to its non-use [90,91,92,93]. Freyer et al. (2017) conducted a phase III multicenter randomized trial involving 38 pediatric oncology centers, in which they analyzed the efficacy of sodium thiosulphate administered 6 h after chemotherapy with cisplatin. The trial showed a lower incidence of hearing damage in patients who received sodium thiosulphate without significant side effects [92]. Brock et al. (2018) also studied the efficacy of sodium thiosulphate administrated 6 h after chemotherapy with cisplatin in another multicenter randomized trial involving pediatric patients with standard-risk hepatoblastoma; they reported lower incidence of cisplatin-induced hearing loss among children who received sodium thiosulphate [93]. Although sodium thiosulphate at present has not become part of common clinical practice, its use appears to promise encouraging progress in the protection of hearing damage from platinum compounds. Currently, it is used in the management of acute cyanide poisoning in association with sodium nitrite.

D-Methionine acts with a mechanism similar to the previously mentioned substances and has been studied as an otoprotective agent not only against platinum derivatives but also against other drugs with ototoxic action, showing good efficacy in preclinical animal studies, although human efficacy data are not yet available [94].

The systemic administration of these compounds, however, could cause a reduced therapeutic response to chemotherapy. Consequently, in recent years, some studies have placed their attention on substances that can be administered locally into the ear. Among these compounds there is *N*-acetylcysteine, which can be administered trans-tympanic and has shown good ear protection capacity [95]. The administration of steroids by the intratympanic route and vitamin E is also effective in protecting against hearing loss [95].

Considering that the main limitations for cisplatin treatment are chemoresistance and side effects, recently two different polyphenols, curcumin and ferulic acid, have been studied as adjuvant chemotherapeutics. Thanks to their biphasic antioxidant activity in normal cells undergoing stressful conditions, such as the hair cells, as well as pro-oxidant activity in cancer cells, these molecules are promising tools in counteracting chemoresistence and ototoxicity [38]. Taken together, these data suggest that the molecular mechanism and the hormetic properties of several molecular candidates for otoprotection need to be carefully examined in pre-clinical studies and clinical trials for a personalized therapeutic approach.

In 2017, Crabb et al. analyzed the possible use of aspirin as an otoprotective drug against cisplatin; their hypothesis was based on the fact that aspirin protected from hearing damage caused by aminoglycosides [96]. However, their study showed no efficacy in the use of aspirin during cisplatin therapy.

Although many studies are focusing on the research of substances with otoprotective function, at present, none of them have shown real efficacy and safety against hearing loss caused by platinum compounds [97].

## 6. Conclusions

Platinum compounds represent a cornerstone in the treatment of many different childhood cancer types, and their use has dramatically improved survival. However, they can determine toxic effects including hearing damage, which is an important problem for patients in the pediatric age group. The knowledge available to date has highlighted the need to carry out a careful audiological follow-up in survivors of childhood cancer that have undergone treatment with platinum compounds. The search for agents that can reduce hearing damage derived from platinum compounds represents a challenge that is still open, and its victory would allow the achievement of significant improvements in the quality of life in this group of patients.

## Figures and Tables

**Table 1:** Audiological tests and ages of application.

Age	Tests
Children younger 7–8 months and not cooperative patients	OAEs measurement
	ABR
Children between ages of 7–8 months and 24–30 months	Visual reinforcement audiometry
Children aged 24 months to 5 or 6 years old	Conditioned play audiometry
Children over 7–8 years old	Pure tone audiometry

## References

[1] Knight K.R., Kraemer D.F., Neuwelt E.A. (2005). Ototoxicity in children receiving platinum chemotherapy: Underestimating a commonly occurring toxicity that may influence academic and social development. J. Clin. Oncol..

[2] Van den Berg H., Van den Anker J.N., Beijnen J.H. (2012). Cytostatic drugs in infants: A review on pharmacokinetic data in infants. Cancer Treat Rev..

[3] Ruggiero A., Trombatore G., Triarico S., Arena R., Ferrara P., Scalzone M., Pierri F., Riccardi R. (2013). Platinum compounds in children with cancer: Toxicity and clinical management. Anticancer Drugs.

[4] Boulikas T., Pantos A., Bellis E., Petros C. (2007). Designing platinum compounds in cancer: Structures and mechanisms. Cancer Ther..

[5] O’Dwyer P.J., Stevenson J.P., Johnson S.W. (2000). Clinical pharmacokinetics and administration of established platinum drugs. Drugs.

[6] Lokich J., Anderson N. (1998). Carboplatin versus cisplatin in solid tumors: An analysis of the literature. Ann. Oncol..

[7] Peng B., English M.W., Boddy A.V., Price L., Wyllie R., Pearson A.D.J., Tilby M.J., Newell D.R. (1997). Cisplatin pharmacokinetics in children with cancer. Eur. J. Cancer.

[8] Go R.S., Adjei A.A. (1999). Review of the comparative pharmacology and clinical activity of cisplatin and carboplatin. J. Clin. Oncol..

[9] Breglio A.M., Rusheen A.E., Shide E.D., Fernandez K.A., Spielbauer K.K., McLachlin K.M., Hall M.D., Amable L., Cunningham L.L. (2017). Cisplatin is retained in the cochlea indefinitely following chemotherapy. Nat. Commun..

[10] UpToDate Cisplatin: Drug Information. http://www.uptodate.com.

[11] Pussegoda K., Ross C.J., Visscher H., Yazdanpanah M., Brooks B., Rassekh S.R., Zada Y.F., Dubé M.P., Carleton B.C., Hayden M.R. (2013). Replication of TPMT and ABCC3 genetic variants highly associated with cisplatin-induced hearing loss in children. Clin. Pharmacol. Ther..

[12] Nakamura T., Yonezawa A., Hashimoto S., Katsura T., Inui K. (2010). Disruption of multidrug and toxin extrusion MATE1 potentiates cisplatin-induced nephrotoxicity. Biochem. Pharmacol..

[13] Kanat O., Ertas H., Caner B. (2017). Platinum-induced neurotoxicity: A review of possible mechanisms. World J. Clin. Oncol..

[14] Knox R.J., Friedlos F., Lydall D.A., Roberts J.J. (1986). Mechanism of cytotoxicity of anticancer platinum drugs: Evidence that cis-diam-minedi-chloroplatinum (II) and cis-diammine (1,1-cyclobutane-diacarbo-xylate) platinum (II) differ only in kinetics of their interaction with DNA. Cancer Res..

[15] Micetich K.C., Barnes D., Erickson L.C. (1985). A comparative study of the cytotoxicity and DNA-damaging effects of cis-(diammino) (1,1- cyclobutanedicarboxylato)-platinum (II) and cis-diamminedichloroplatinum (II) on L1210 cells. Cancer Res..

[16] UpToDate Carboplatin: Drug information. http://www.uptodate.com.

[17] Veal G.J., Errington J., Tilby M.J., Pearson A.D., Foot A.B., McDowell H., Ellershaw C., Pizer B., Nowell G.M., Pearson D.G. (2007). Adaptive dosing and platinum–DNA adduct formation in children receiving high-dose carboplatin for the treatment of solid tumours. Br. J. Cancer.

[18] Lebwohl D., Canetta R. (1998). Clinical development of platinum complexes in cancer therapy: An historical perspective and an update. Eur. J. Cancer.

[19] Kweekel D.M., Gelderblom H., Guchelaar H.J. (2005). Pharmacology of oxaliplatin and the use of pharmacogenomics to individualize therapy. Cancer Treat. Rev..

[20] Di Francesco A.M., Ruggiero A., Riccardi R. (2002). Cellular and molecular aspects of drugs of the future: Oxaliplatin. Cell. Mol. Life Sci..

[21] Page J.D., Husain I., Sancar A., Chaney S.G. (1990). Effect of the diaminocyclohexane carrier ligand on platinum adduct formation, repair, and lethality. Biochemistry.

[22] Fink D., Nebel S., Aebi S., Zheng H., Cenni B., Nehme A., Christen R.D., Howell S.B. (1996). The role of DNA mismatch repair in platinum drug resistance. Cancer Res..

[23] Vaisman A., Varchenko M., Umar A., Kunkel T.A., Risinger J.I., Barrett J.C., Hamilton T.C., Chaney S.G. (1998). The role of hMLH1, hMSH3, and hMSH6 defects in cisplatin and oxaliplatin resistance: Correlation with replicative bypass of platinum–DNA adducts. Cancer Res..

[24] Raymond E., Lawrence R., Izbicka E., Faivre S., Von Hoff D.D. (1998). Activity of oxaliplatin against human tumor colony forming units. Clin. Cancer Res..

[25] Riccardi A., Ferlini C., Meco D., Mastrangelo R., Scambia G., Riccardi R. (1999). Antitumor activity of oxaliplatin in neuroblastoma cell lines. Eur. J. Cancer.

[26] Bass J.K., Knight K.R., Yock T.I., Chang K.W., Cipkala D., Grewal S.S. (2016). Evaluation and management of hearing loss in survivors of childhood and adolescent cancers: A report from the children’s oncology group. Pediatr. Blood Cancer.

[27] Bertolini P., Lassalle M., Mercier G., Raquin M.A., Izzi G., Corradini N., Hartmann O. (2004). Platinum compound-related ototoxicity in children: Long-term follow-up reveals continuous worsening of hearingloss. J. Pediatr. Hematol. Oncol..

[28] Hua C., Bass J.K., Khan R., Kun L.E., Merchant T.E. (2008). Hearing loss after radiotherapy for Pediatric brain tumors: Effect of cochlear dose. Int. J. Radiat. Oncol. Biol. Phys..

[29] Kolinsky D.C., Hayashi S.S., Karzon R., Mao J., Hayashi R.J. (2010). Late onset hearing loss: A significant complication of cancer survivors treated with cisplatin containing chemotherapy regimens. J. Pediatr. Hematol. Oncol..

[30] Rybak L.P., Whitworth C.A., Mukherjea D., Ramkumar V. (2007). Mechanisms of cisplatin-induced ototoxicity and prevention. Hear. Res..

[31] Langer T., Zehnhoff-Dinnesen A., Radtke S., Meitert J., Zolk O. (2013). Understanding platinum-induced ototoxicity. Trends Pharmacol. Sci..

[32] Brock P.R., Knight K.R., Freyer D.R., Campbell K.C., Steyger P.S., Blakley B.W., Rassekh S.R., Chang K.W., Fligor B.J., Rajput K. (2012). Platinum-induced ototoxicity in children: A consensus review on mechanisms, predisposition, and protection, including a new international society of Pediatric oncology Boston ototoxicity scale. J. Clin. Oncol..

[33] Kopke R.D., Liu W., Gabaizadeh R., Jacono A., Feghali J., Spray D., Garcia P., Steinman H., Malgrange B., Ruben R.J. (1997). Use of organotypic cultures of Corti’s organ to study the protective effects of antioxidant molecules on cisplatin-induced damage of auditory hair cells. Am. J. Otol..

[34] Dehne N., Lautermann J., Petrat F., Rauen U., de Groot H. (2001). Cisplatin ototoxicity: Involvement of iron and enhanced formation of superoxide anion radicals. Toxicol. Appl. Pharmacol..

[35] Bánfi B., Malgrange B., Knisz J., Steger K., Dubois-Dauphin M., Krause K.H. (2004). NOX3, a superoxide-generating NADPH oxidase of the inner ear. J. Biol. Chem..

[36] Rybak L.P., Mukherjea D., Ramkumar V. (2019). Mechanisms of cisplatin-induced ototoxicity and prevention. Seminars in Hearing.

[37] Rybak L.P., Husain K., Morris C., Whitworth C., Somani S. (2000). Effect of protective agents against cisplatin ototoxicity. Am. J. Otol..

[38] Paciello F., Fetoni A., Mezzogori D., Rolesi R., Di Pino A., Paludetti G., Grassi G., Troiani D. (2020). The dual role of curcumin and ferulic acid in counteracting chemoresistance and cisplatin-induced ototoxicity. Sci. Rep..

[39] Riedemann L., Lanvers C., Deuster D., Peters U., Boos J., Jürgens H., am Zehnhoff-Dinnesen A. (2008). Megalin genetic polymorphisms and individual sensitivity to the ototoxic effect of cisplatin. Pharm. J..

[40] Xu X., Ren H., Zhou B., Zhao Y., Yuan R., Ma R., Zhou H., Liu Z. (2012). Prediction of copper transport protein 1 (CTR1) genotype on severe cisplatin induced toxicity in non-small cell lung cancer (NSCLC) patients. Lung Cancer.

[41] Brusilow S.W., Gordes E. (1973). The mutual independence of the endolymphatic potential and the concentrations of sodium and potassium in endolymph. J. Clin. Investig..

[42] Altura B.M., Altura B.T., Gebrewold A., Ising H., Günther T. (1984). Magnesium deficiency and hypertension: Correlation between magnesium deficient diets and microcirculatory changes in situ. Science.

[43] Brown R. (1975). Ethacrinic acid and furosemide: Possible cochlear sites and mechanisms of ototoxic action. Mednikon.

[44] Joachims Z., Babisch W., Ising H., Günther T., Handrock M. (1983). Dependence of noise induced hearing loss on perilymph magnesim concentration. J. Acoust. Soc. Am..

[45] Grewal S., Merchant T., Reymond R., McInerney M., Hodge C., Shearer P. (2010). Auditory late effects of childhood cancer therapy: A report from the children’s oncology group. Pediatrics.

[46] Huang R.S., Kistner E.O., Bleibel W.K., Shukla S.J., Dolan M.E. (2007). Effect of population and gender on chemotherapeutic agent-induced cytotoxicity. Mol. Cancer Ther..

[47] Martindale J.L., Holbrook N.J. (2002). Cellular response to oxidative stress: Signaling for suicide and survival. J. Cell Physiol..

[48] Faragher A.J., Fry A.M. (2003). Nek2A kinase stimulates centrosome disjunction and is required for formation of bipolar mitotic spindles. Mol. Biol. Cell.

[49] Lin C.P., Liu J.D., Chow J.M., Liu C.R., Liu H.E. (2007). Small-molecule c-Myc inhibitor, 10058-F4, inhibits proliferation, downregulates human telomerase reverse transcriptase and enhances chemosensitivity in human hepatocellular carcinoma cells. Anticancer Drugs.

[50] Peters U., Preisler-Adams S., Hebeisen A., Hahn M., Seifert E., Lanvers C., Heinecke A., Horst J., Jürgens H., Lamprecht-Dinnesen A. (2000). Glutathione S-transferase genetic polymorphisms and individual sensitivity to the ototoxic effect of cisplatin. Anticancer Drugs.

[51] Oldenburg J., Kraggerud S.M., Cvancarova M., Lothe R.A., Fossa S.D. (2007). Cisplatin-induced long-term hearing impairment is associated with specific glutathione S-transferase genotypes in testicular cancer survivors. J. Clin. Oncol..

[52] Peters U., Preisler-Adams S., Lanvers-Kaminsky C., Jürgens H., Lamprecht-Dinnesen A. (2003). Sequence variations of mitochondrial DNA and individual sensitivity to the ototoxic effect of cisplatin. Anticancer Res..

[53] Knoll C., Smith R.J., Shores C., Blatt J. (2009). Hearing genes and cisplatin deafness: A pilot study. Laryngoscope.

[54] Clemens E., Broer L., Langer T., Uitterlinden A.G., de Vries A.C.H., van Grotel M., Pluijm S.F.M., Binder H., Byrne J., Broeder E.V.D. (2020). Genetic variation of cisplatin-induced ototoxicity in non-cranial-irradiated pediatric patients using a candidate gene approach: The International PanCareLIFE Study. Pharm. J..

[55] Li Y., Womer R.B., Silber J.H. (2004). Predicting cisplatin ototoxicity in children: The infl uence of age and the cumulative dose. Eur. J. Cancer.

[56] Lanvers-Kaminsky C., Zehnhoff-Dinnesen A.A., Parfitt R., Ciarimboli G. (2017). Drug-induced ototoxicity: Mechanisms, pharmacogenetics, and protective strategies. Clin. Pharmacol. Ther..

[57] Shi X., Nuttall A.L. (2003). Upregulated iNOS and oxidative damage to the cochlear stria vascularis due to noise stress. Brain Res..

[58] Ding D., Allman B.L., Salvi R. (2012). Ototoxic characteristics of platinum antitumor drugs. Anat. Rec..

[59] Gratton M.A., Salvi R.J., Kamen B.A., Saunders S.S. (1990). Interaction of cisplatin and noise on the peripheral auditory system. Hear. Res..

[60] Rabiço-Costa D., Gil-da-Costa M.J., Barbosa J.P., Bom-Sucesso M., Spratley J. (2020). Platinum-drugs ototoxicity in Pediatric patients with brain tumors: A 10-year review. J. Pediatr. Hematol. Oncol..

[61] Khan A., Budnick A., Barnea D., Feldman D.R., Oeffinger K.C., Tonorezos E.S. (2018). Hearing loss in adult survivors of childhood cancer treated with radiotherapy. Child. Basel.

[62] Bass J.K., Huang J., Onar-Thomas A., Chang K.W., Bhagat S.P., Chintagumpala M., Bartels U., Gururangan S., Hassall T., Heath J.A. (2014). Concordance between the Chang and the International Society of Pediatric Oncology (SIOP) Ototoxicity Grading Scales in patients treated with cisplatin for medulloblastoma. Pediatr. Blood Cancer.

[63] Delpire E., Lu J., England R., Dull C., Thorne T. (1999). Deafness and imbalance associated with inactivation of the secretory Na-K-2Cl co-transporter. Nat. Genet..

[64] Farinetti A., Raji A., Wu H., Wanna B., Vincent C. (2018). International consensus (ICON) on audiological assessment of hearing loss in children. Eur. Ann. Otorhinolaryngol. Head Neck Dis..

[65] Anastasio A.R., Radael R.D., Cavalcante J.M., Hatzopoulos S. (2012). A report of extended high frequency audiometry thresholds in school-age children with no hearing complaints. Audiol. Res..

[66] Hunter L.L., Monson B.B., Moore D.R., Dhar S., Wright B.A., Munro K.J., Zadeh L.M., Blankenship C.M., Stiepan S.M., Siegel J.H. (2020). Extended High Frequency Hearing and Speech Perception Implications in Adults and Children. Hear. Res..

[67] Durrant J.D., Campbell K., Fausti S., Guthrie O., Jacobson G., Lonsbury-Martin B.L., Poling G. (2009). American Academy of Audiology Position Statement and Clinical Practice Guidelines: Ototoxicity Monitoring. http://www.audiology.org/resources/documentlibrary/Documents/OtoMonGuidelines.

[68] Fausti S.A., Thompson M., Williams J., Bouchard K.R., Farrer S.M., Heifer K.S., McHaney V.A., Tysklind J.M., Durrant J., Fowler C. (1994). Audiologic Management of Individuals Receiving Cochleotoxic Drug Therapy, American Speech-Language-Hearing Association. http://www.asha.org/policy/GL1994-00003.htm.

[69] Knight K.R., Chen L., Freyer D., Aplenc R., Bancroft M., Bliss B., Dang H., Gillmeister B., Hendershot E., Kraemer D.F. (2017). Group-wide, prospective study of ototoxicity assessment in children receiving cisplatin chemotherapy (ACCL05C1): A report from the children’s oncology group. J. Clin. Oncol..

[70] King K.A., Brewer C.C. (2018). Clinical trials, ototoxicity grading scales, and the audiologist’s role in therapeutic decision making. Int. J. Audiol..

[71] Clemens E., van den Heuvel-Eibrink M.M., Mulder R.L., Kremer L.C.M., Hudson M.M., Skinner R., Constine L.S., Bass J.K., Kuehni C.E., Langer T. (2019). International guideline harmonization group ototoxicity group. Recommendations for ototoxicity surveillance for childhood, adolescent, and young adult cancer survivors: A report from the international late effects of childhood cancer guideline harmonization group in collaboration with the pancare consortium. Lancet Oncol..

[72] Lewis M.J., Du Bois S.G., Fligor B., Li X., Goorin A., Grier H.E. (2009). Ototoxicity in children treated for osteosarcoma. Pediatr. Blood Cancer.

[73] Peleva E., Emami N., Alzahrani M., Bezdjian A., Gurberg J., Carret A.S., Daniel S.J. (2014). Incidence of platinum-induced ototoxicity in Pediatric patients in Quebec. Pediatr. Blood Cancer.

[74] Stöhr W., Langer T., Kremers A., Bielack S., Lamprecht-Dinnesen A., Frey E., Beck J.D. (2005). german late effects working group in the german society of Pediatric oncology and hematology. Cisplatin-induced ototoxicity in osteosarcoma patients: A report from the late effects surveillance system. Cancer Investig..

[75] Coradini P.P., Cigana L., Selistre S.G., Rosito L.S., Brunetto A.L. (2007). Ototoxicity from cisplatin therapy in childhood cancer. J. Pediatr. Hematol. Oncol..

[76] Fetoni A.R., Ruggiero A., Lucidi D., De Corso E., Sergi B., Conti G., Paludetti G. (2016). Audiological monitoring in children treated with platinum chemotherapy. Audiol. Neurootol..

[77] Stachler R.J., Chandrasekhar S.S., Archer S.M., Rosenfeld R.M., Schwartz S.R., Barrs D.M., Brown S.R., Fife T.D., Ford P., Ganiats T.G. (2012). American academy of otolaryngology-head and neck surgery. Clinical practice guideline: Sudden hearing loss. Otolaryngol. Head Neck Surg..

[78] NICE: London (2009). National Institute for Health and Care Excellence. Cochlear Implants for Children and Adults with Severe to Profound Deafness. https://www.nice.org.uk/guidance/ta166.

[79] New York State Department of Health (2007). Clinical Practice Guideline: Report of the Recommendations. Hearing Loss: Assessment and Intervention for Young Children (Age 0–3 Years). https://www.health.ny.gov/community/infants_children/early_intervention/docs/guidelines_hearing_loss_recommendations.pdf.

[80] American Association of Audiology (2013). Clinical Practice Guidelines on Pediatric Amplification. http://galster.net/wp-content/uploads/2013/07/AAA-2013-Pediatric-Amp-Guidelines.pdf.

[81] Audiology Australia (2013). Professional Practice Standards: Audiological Rehabilitation. https://audiology.asn.au/Tenant/C0000013/Position%20Papers/Member%20Resources/Clinical%20Standards%20partb%20-%20whole%20document%20July13%201.

[82] Fetoni A.R., Paciello F., Mezzogori D., Rolesi R., Eramo S.L., Paludetti G., Troiani D. (2015). Molecular targets for anticancer redox chemotherapy and cisplatin-induced ototoxicity: The role of curcumin on pSTAT3 and Nrf-2 signalling. Br. J. Cancer.

[83] Fetoni A.R., Eramo S.L., Paciello F., Rolesi R., Podda M.V., Troiani D., Paludetti G. (2014). Curcuma longa (curcumin) decreases in vivo cisplatin-induced ototoxicity through heme oxygenase-1 induction. Otol. Neurotol..

[84] Fetoni A.R., Sergi B., Ferraresi A., Paludetti G., Troiani D. (2004). Protective effects of alpha-tocopherol and tiopronin against cisplatin-induced ototoxicity. Acta Otolaryngol..

[85] Lafay-Cousin L., Purdy E., Huang A., Cushing S.L., Papaioannou V., Nettel-Aguirre A., Bouffet E. (2013). Early cisplatin induced ototoxicity profile may predict the need for hearing support in children with medulloblastoma. Pediatr. Blood Cancer.

[86] Van As J.W., van den Berg H., van Dalen E.C. (2012). Medical interventions for the prevention of platinum-induced hearing loss in children with cancer. Cochrane Database Syst. Rev..

[87] Fouladi M., Chintagumpala M., Ashley D., Kellie S., Gururangan S., Hassall T., Gronewold L., Stewart C.F., Wallace D., Broniscer A. (2008). Amifostine protects against cisplatin-induced ototoxicity in children with average-risk medulloblastoma. J. Clin. Oncol..

[88] Katzenstein H.M., Chang K.W., Krailo M., Chen Z., Finegold M.J., Rowland J., Reynolds M., Pappo A., London W.B., Malogolowkin M. (2009). Children’s Oncology Group. Amifostine does not prevent platinum induced hearing loss associated with the treatment of children with hepatoblastoma: A report of the Intergroup Hepatoblastoma Study P9645 as a part of the Children’s Oncology Group. Cancer.

[89] Neuwelt E.A., Gilmer-Knight K., Lacy C., Nicholson H.S., Kraemer D.F., Doolittle N.D., Hornig G.W., Muldoon L.L. (2006). Toxicity profile of delayed high dose sodium thiosulfate in children treated with carboplatin in conjunction with blood–brain barrier disruption. Pediatr. Blood Cancer.

[90] Duval M., Daniel S. (2012). Meta-analysis of the efficacy of amifostine in the prevention of cisplatin ototoxicity. J. Otolaryngol. Head Neck Surg..

[91] Doolittle N.D., Muldoon L.L., Brummett R.E., Tyson R.M., Lacy C., Bubalo J.S., Kraemer D.F., Heinrich M.C., Henry J.A., Neuwelt E.A. (2001). Delayed sodium thiosulfate as an otoprotectant against carboplatin-induced hearing loss in patients with malignant brain tumors. Clin. Cancer Res..

[92] Freyer D.R., Chen L., Krailo M.D., Knight K., Villaluna D., Bliss B., Pollock B.H., Ramdas J., Lange B., Van Hoff D. (2017). Effects of sodium thiosulfate versus observation on development of cisplatin-induced hearing loss in children with cancer (ACCL0431): A multicentre, randomised, controlled, open-label, phase 3 trial. Lancet Oncol..

[93] Brock P.R., Maibach R., Childs M., Rajput K., Roebuck D., Sullivan M.J., Laithier V., Ronghe M., Dall’Igna P., Hiyama E. (2018). Sodium thiosulfate for protection from cisplatin-induced hearing loss. N. Engl. J. Med..

[94] Campbell K.C., Meech R.P., Klemens J.J., Gerberi M.T., Dyrstad S.S., Larsen D.L., Mitchell D.L., El-Azizi M., Verhulst S.J., Hughes L.F. (2007). Prevention of noise- and drug-induced hearing loss with D-methionine. Hear. Res..

[95] Riga M.G., Chelis L., Kakolyris S., Papadopoulos S., Stathakidou S., Chamalidou E., Xenidis N., Amarantidis K., Dimopoulos P., Danielides V. (2018). Transtympanic injections of N-acetylcysteine for the prevention of cisplatin-induced ototoxicity: A feasible method with promising efficacy. Am. J. Clin. Oncol..

[96] Crabb S.J., Martin K., Abab J., Ratcliffe I., Thornton R., Lineton B., Ellis M., Moody R., Stanton L., Galanopoulou A. (2017). COAST (Cisplatin ototoxicity attenuated by aspirin trial): A phase II double-blind, randomised controlled trial to establish if aspirin reduces cisplatin induced hearing-loss. Eur. J. Cancer.

[97] Freyer D.R., Brock P., Knight K., Reaman G., Cabral S., Robinson P.D., Sung L. (2019). Interventions for cisplatin-induced hearing loss in children and adolescents with cancer. Lancet Child. Adolesc. Health.

